# Association Between *agr* Type, Virulence Factors, Biofilm Formation and Antibiotic Resistance of *Staphylococcus aureus* Isolates From Pork Production

**DOI:** 10.3389/fmicb.2018.01876

**Published:** 2018-08-20

**Authors:** Yang Zhang, Dongyang Xu, Lei Shi, Rujian Cai, Chunling Li, He Yan

**Affiliations:** ^1^School of Food Science and Engineering, South China University of Technology, Guangzhou, China; ^2^Institute of Genomics, Huaqiao University, Xiamen, China; ^3^State Key Laboratory of Food Safely Technology for Meat Products, Xiamen, China; ^4^Institute of Animal Health, Guangdong Academy of Agricultural Sciences, Guangzhou, China

**Keywords:** *Staphylococcus aureus*, *agr* typing, biofilm formation, virulence gene, antibiotic resistance, pork production

## Abstract

Livestock-associated *Staphylococcus aureus* colonization and/or infections exist in pigs and people in frequent contact with pigs. In this study, a total of 130 *S. aureus* isolates obtained from different stages of pork production were subjected to antimicrobial susceptibility, biofilm formation, as well as PCR screening to identify virulence genes, and the accessory gene regulator alleles (*agr*). Among all 130 *S. aureus* isolates, 109 (83.8%, 109/130) isolates were positive for *agr*. All swine farms isolates belonged to *agr* IV, whereas *S. aureus* isolated from slaughterhouse and retail indicated diverse *agr* types. All isolates exhibited biofilm formation ability, and raw meat isolates (belonging to *agr* I) exhibited a greater ability to form strong biofilms than swine farms isolates (belonging to *agr* IV). *agr*-positive isolates were associated with more virulence genes than *agr*-negative isolates. Most biofilm-producing isolates were positive for microbial surface component recognizing adhesive matrix molecule (MSCRAMM), capsule type and *ica* group genes. The results illustrate a significant association between the prevalence rate of MSCRAMM, capsule type and *ica* group genes among isolates producing weak, moderate and strong biofilms. The high prevalence of resistance to ciprofloxacin, gentamicin, tetracycline, clarithromycin, clindamycin, and trimethoprim-sulfamethoxazole were mainly observed in moderate and weak biofilm producers. Our findings indicate that *S. aureus* isolates from pork production displayed diverse molecular ecology.

## Introduction

*Staphylococcus aureus* is an important zoonotic pathogen that is responsible for a variety of infectious diseases characterized by septicemia and sepsis (Crombe et al., 2013; Song et al., 2015). China is one of the world’s largest pork producers with more than 470 million pigs, accounting for ∼50% of the total numbers in the world (Krishnasamy et al., 2015). Consecutively, several reports suggested transmission between pigs and humans causing livestock-associated *S. aureus* (LA-SA) colonization in 23–45% of pig-farmers (Voss et al., 2005; Huijsdens et al., 2006; Smith et al., 2008) and 4.6% of pig-care veterinarians (Wulf et al., 2006). The “One Health” concept recognizes that human health or livestock or wildlife health are interconnected and bound to the animal-human-ecosystems in which they (co)exist. Occupational exposure to swine has been associated with increased *Staphylococcus aureus* carriage, and increased risk of colonization and infections of different hosts (Witte et al., 2007; Graveland et al., 2011; Price et al., 2012; Kock et al., 2013; Ge et al., 2017; Davis et al., 2018). The risk of zoonotic transmission to humans demands our deep understanding of *S. aureus* contamination and ecology in the swine production.

The rapid development of resistance to multiple antimicrobial agents increases the difficulty treating *S. aureus* infections and biofilm production facilitate this organism to survive in the presence of antibiotics (Dhanawade et al., 2010; Bhattacharya et al., 2018). Several studies have demonstrated that low doses of certain antibiotics could induce biofilm formation, indicating that biofilm regulation might be involved in the global response to external stresses, including antibiotics (Hoffman et al., 2005; Kaplan, 2011). Previous studies regarding quantitative correlation between biofilm formation and antibiotics resistance have yielded different results. For example, Neopane et al. (2018) concluded that the biofilm-positive strains have a higher tendency to exhibit multidrug resistance and methicillin resistance compared to biofilm-negative strains, while Eyoh et al. (2014) indicated that there was no significant difference in the percentage of multi-drug-resistance (MDR) among biofilm producers and non-biofilm formers for both medical and non-medical personnel.

*Staphylococcus aureus* produces a wide variety of protein toxins, such as exfoliative toxins, Panton-Valentine leukocidin, hemolysins, enterotoxins, and toxic shock syndrome toxin. Among the large array of *S. aureus* virulence factors the MSCRAMMs (microbial surface component recognizing adhesive matrix molecules) includes different adhesins, which are essential for initial stages of infection (Magro et al., 2017). MSCRAMMs, which includes fibronectin binding proteins (FnbA and FnbB), fibrinogen binding proteins (ClfA, ClfB and Efb), capsule proteins (Capsule type 5 and 8) and collagen binding proteins (Cna), can bind to a variety of mammalian extracellular proteins and abiotic surfaces (Donlan, 2002). Furthermore, the formation of a highly organized multicellular biofilm is related to the polysaccharide intercellular adhesin (PIA) production, which is controlled by the *ica* operon (Cramton et al., 1999). Therefore, the numbers and combinations of toxin genes may contribute to the pathogenicity of *S. aureus*.

While previous studies have documented the prevalence of *S. aureus* isolates in bovine mastitis (Fluit, 2012; Snel et al., 2015; Artursson et al., 2016; Kot et al., 2016; Magro et al., 2017), there is a lack of data regarding the prevalence and characterization of *S. aureus* in pork production. A thorough understanding of the correlation between the observed polymorphism in genotype and virulence, and the diversity in production practices is important for targeted mitigation. In this study, an extensive study was conducted involving systematic sampling of three commercial swine farms, a contracted slaughterhouse for the designated farms, and a retail market in Xiamen, China to profile *S. aureus* isolates along the production, processing and retail chain. The data enabled tracking of the spread of *S. aureus* from pork production and a better understanding of the evolution of *S. aureus*.

## Materials and Methods

### Bacterial Strains and Antibiotic Susceptibility

From September – December 2014, three commercial swine farms with > 5000 pigs, one large slaughterhouse and several terminal markets were selected from Xiamen City, People’s Republic of China, and 501 samples were collected from these places for *S. aureus* isolation. Pigs were born and raised in these three commercial swine farms with distance for more than 25 km from each other and then were sent to the slaughterhouse. These three swine farms and the slaughterhouse were vertically integrated pork processing plant, meaning pigs originated from these three swine farms contracted to sell hogs exclusively to the slaughterhouse. However, terminal samples from the markets did not totally originate from the slaughterhouse tested in the present study.

Briefly, a total of 501 non-duplicate samples were collected from the pork industry, including three commercial swine farms (sty door and soil, *n* = 71; nasal swabs, *n* = 97), one slaughterhouse (pork, *n* = 173), and terminal markets (pork, *n* = 160). Isolation and identification of *S. aureus* were performed according to China’s National Technical Standard GB4789.10-2010 and the special gene *nuc* was targeted by PCR for identifying *S. aureus* (Brakstad et al., 1992). Contamination with *S. aureus* was detected in 26.0% (130/501) of the total samples, and the prevalence of *S. aureus* was highest in the slaughterhouse (35.8%, 62/173) followed by the market (24.4%, 39/160) and the farm (17.3%, 29/168).

These isolates were assessed for antimicrobial susceptibility by the Kirby-Bauer disk diffusion method described by the Clinical and Laboratory Standards Institute (CLSI, 2012). The antibiotic disks used (Hangzhou Microbial Reagent Co., Ltd., Hangzhou) included ciprofloxacin (5 μg), penicillin (10 μg), gentamicin (10 μg), tetracycline (30 μg), clarithromycin (15 μg), clindamycin (2 μg), chloramphenicol (30 μg), sulfamethoxazole-trimethoprim (25 μg), nitrofurantoin (30 μg), rifampin (5 μg), cephalothin (30 μg), minocycline (30 μg), cefoxitin (30 μg) and oxacillin (1 μg).

### *agr* Genotyping

Bacterial genomic DNA template was extracted from the isolates by a commercial DNA extraction kit (Biomed, Beijing, China). The *agr* types (I–IV) were determined by a multiplex PCR assay as described by Gilot et al. (2002). In brief, multiplex PCR was performed with the following primers: Pan (5′-ATG CAC ATG GTG CAC ATG C-3′), agr1 (5′-GTC ACA AGT ACT ATA AGC TGC GAT-3′), agr2 (5′-TAT TAC TAA TTG AAA AGT GGC CAT AGC-3′), agr3 (5′-GTA ATG TAA TAG CTT GTA TAA TAA TAC CCA G-3′) and agr4 (5′-CGA TAA TGC CGT AAT ACC CG-3′). These primers yield a PCR product of 441, 575, 323, or 659 bp corresponding to *agr* group I, II, III, and IV, respectively. Each assay contained 2 μL of prepared DNA template, 2.5 μL of 10× Easy Taq Buffer [Takara Biomedical Technology (Beijing) Co., Ltd, China], 1 μL of 10 mM deoxynucleotide triphosphate [Takara Biomedical Technology (Beijing) Co., Ltd, China], 1 μL of upstream and downstream primers (10 μM), and 0.125 μL of DNA polymerase (5 U/μL) [Takara Biomedical Technology (Beijing) Co., Ltd, China], and the final system volume was adjusted to 25 μL with sterile ultrapure water. The PCR conditions were as follows: 1 cycle at 94°C for 5 min; 26 cycles at 94°C for 30 s, 55°C for 30 s, and 72°C for 1 min; and finally 1 cycle at 72°C for 10 min. All PCR products were analyzed by electrophoresis on a 1.5% (w/v) agarose gel.

### Identification of Virulence Determinants

The nucleotide sequences of all PCR primers used in this study and their respective amplified products and specific Tm (°C) are listed in **Table 1**. All the oligonucleotide primers were synthesized by Sangon Biotech (Shanghai, China). Each assay contained 1 μL of prepared DNA template, 2.5 μL of 10× Easy Taq Buffer [Takara Biomedical Technology (Beijing) Co., Ltd, China], 1 μL of 10 mM deoxynucleotide triphosphate [Takara Biomedical Technology (Beijing) Co., Ltd, China], 1 μL of upstream and downstream primers (10 μM), and 0.125 μL of DNA polymerase (5 U/μL) [Takara Biomedical Technology (Beijing) Co., Ltd, China], and the final system volume was adjusted to 25 μL with sterile ultrapure water. The PCR conditions were as follows: an initial denaturation at 95°C for 5 min; 30 cycles of 95°C for 30 s, specific Tm for 30 s, and 72°C for 40–90 s depending on the PCR product length; and a final extension at 72°C for 10 min. Sequencing of the extracted PCR product was performed by Beijing Genomics Institute (Shenzhen, China) and the data were analyzed with the GenBank database using the BLAST algorithm at the National Center for Biotechnology Information web site^1^.

**Table 1:** Target genes, putative function of encoded protein, primer sequence, and PCR conditions.

Target gene	Primer name	Putative function of encoded protein	Primer sequence (5′—3′)	Product size (bp)	Tm (°C)	Reference
*clfA*	clfA-F	Encoding Clumping factor, ClfA	AAAACACGCAATTCGGAAAA	855	53	Ote et al., 2011
	clfA-R		GCAGTTGAAGTTACACCATTTAAGT			
*clfB*	clfB-F	Encoding Clumping factor, ClfB	TGTCGAATAAGCAGAATAAG	505	49	Ote et al., 2011
	clfB-R		GGTGATGATTGTGGTAAATC			
*bbp*	bbp-F	Encoding bone sialoprotein-binding protein, Bbp	AACTACATCTAGTACTCAACAACAG	575	55	Tristan et al., 2003
	bbp-R		ATGTGCTTGAATAACACCATCATCT			
*ebpS*	ebpS-F	Encoding cell surface elastin-binding protein	CATCCAGAACCAATCGAAGAC	186	55	Tristan et al., 2003
	ebpS-R		CTTAACAGTTACATCATCATGTTTATCTTTG			
*cna*	cna-F	Encoding collagen-binding protein	GTCAAGCAGTTATTAACACCAGAC	423	55	Tristan et al., 2003
	cna-R		AATCAGTAATTGCACTTTGTCCACTG			
*eno*	eno-F	Encoding laminin binding protein	ACGTGCAGCAGCTGACT	302	55	Tristan et al., 2003
	eno-R		CAACAGCATYCTTCAGTACCTTC			
*fib*	fib-F	Encoding fibrinogen binding protein, Fib	CTACAACTACAATTGCCGTCAACAG	404	55	Tristan et al., 2003
	fib-R		GCTCTTGTAAGACCATTTTCTTCAC			
*fnbA*	fnbA-F	Encoding fibronectin-binding protein A	GTGAAGTTTTAGAAGGTGGAAAGATTAG	643	55	Tristan et al., 2003
	fnbA-R		GCTCTTGTAAGACCATTTTTCTTCAC			
*fnbB*	fnbB-F	Encoding fibronectin-binding protein B	GTAACAGCTAATGGTCGAATTGATACT	524	55	Tristan et al., 2003
	fnbB-R		CAAGTTCGATAGGAGTACTATGTTC			
*cap5*	cap5-F	Encoding CP5 synthesis enzyme	ATGAGGATAGCGATTGAAAA	518	49	Ote et al., 2011
	cap5-R		CGCTTCTTAATCACTTTTGC			
*cap8*	cap8-F	Encoding CP8 synthesis enzyme	ATCGAAGAACATATCCAAGG	834	46	Ote et al., 2011
	cap8-R		TTCATCACCAATACCTTTTA			
*icaA*	icaA-F	Encoding intercellular adhesion protein A	CTTGCTGGCGCAGTCAATAC	178	55	Pereyra et al., 2016
	icaA-R		CCAACATCCAACACATGGCA			
*icaC*	icaC-F	Encoding intercellular adhesion protein C	CTTGGGTATTTGCACGCATT	209	55	Pereyra et al., 2016
	icaC-R		GCAATATCATGCCGACACCT			
*icaD*	icaD-F	Encoding intercellular adhesion protein D	CGCTATATCGTGTGTCTTTTGGA	164	55	Pereyra et al., 2016
	icaD-R		TCGCGAAAATGCCCATAGTT			
*bap*	bap-F	Encoding biofilm-associated protein, Bap	CCCTATATCGAAGGTGTAGAATTGCAC	971	60	Pereyra et al., 2016
	bap-R		GCTGTTGAAGTTAATACTGTACCTGC			
*pvl*	pvl-F	Encoding Panton-Valentine leukocidin	GTCGTTAGGAATAATCACTCC	423	48	Ote et al., 2011
	pvl-R		CCTGTTGATGGACCACTATTAA			
*tst*	tsst-F	Encoding toxic shock syndrome toxin-1	TTTTTTATCGTAAGCCCTTTGTTGC	550	51	Ote et al., 2011
	tsst-R		CACCCGTTTTATCGCTTGAA			
*hla*	hla-F	Encoding alpha-haemolysin precursor	TGCCGCAGATTCTGATATTAA	845	51	Ote et al., 2011
	hla-R		TTTCTGAAGAACGATCTGTCCA			
*hlb*	hlb-F	Encoding beta-haemolysin precursor	GCGGTTGTGGATTCGATAAT	524	50	Ote et al., 2011
	hlb-R		GGCTTTGATTGGGTAATGATC			
*hld*	hld-F	Encoding delta-haemolysin precursor	GGGATGGCTTAATAACTCATACTT	236	48	Ote et al., 2011
	hld-R		CAGAGATGTGATGGAAAATAGTTGA			
*eta*	eta-F	Encoding exfoliative toxin A	TTGTAAAAGGACAAACAAGTGC	544	49.4	Ote et al., 2011
	eta-R		TTCCCAATACCAACACCA			
*etb*	etb-F	Encoding exfoliative toxin B	TTACAAGCAAAAGAATACAGCG	641	50	Ote et al., 2011
	etb-R		GGAAGATTATGTTGTCCGCC			

### Biofilm Formation

Quantification of biofilm formation was performed by spectrophotometry in microplates (Nest Biotechnology Co., Ltd. Wuxi, China) using crystal violet staining as previously described (Pereyra et al., 2016). Briefly, 20 μL of bacterial log phase culture was added to 200 μL of fresh 1% glucose BHI in 96-well flat-bottom microtiter plates. *S. aureus* ATCC25923 (biofilm-forming) and *S. epidermidis* ATCC12228 (not biofilm-forming) were used as positive and negative controls, respectively. BHI without bacteria served as the blank. The plates were incubated at 37°C for 24, 48, and 72 h under aerobic conditions. After each sampling time, wells were washed three times with 300 μL of sterile phosphate-buffered saline (PBS; pH 7.2) and drained by inversion. Subsequently, 200 μL of methanol was added to each well and the plates were dried for 15 min. The adherent cells were stained with 150 μL of 0.1% crystal violet solution for 15 min and then washed twice with sterile water. Bound crystal violet was dissolved by treatment with 150 μL of 95% ethanol for 10 min, and OD_570_ was measured for the stained bacteria and control wells. The experiment was performed in triplicate. An OD_570_ value of 0.3 was taken as the cutoff point to differentiate between biofilm producers and non-biofilm-producer strains [cut-off value (ODc) = average OD of negative control + 3× standard deviation (SD) of negative control] (Pereyra et al., 2016). The quantitative classification of biofilm production based on ODc and average OD values was carried out, resulting in four categories of strains: strong biofilm producers (OD > 4 × ODc), moderate biofilm producers (4 × ODc > OD > 2 × ODc), weak biofilm producers (2 × ODc > OD > ODc), and no biofilm producers (OD < ODc) (Pereyra et al., 2016).

### Growth Rate Analysis

The growth of 12 strong, 12 moderate and 12 weak biofilm formers were measured according to Qi et al. (2016). Briefly, isolates were cultured in BHI agar for 18–24 h and adjusted to 0.5 McFarland units with 0.85% NaCl medium, and diluted 1: 20 in BHI medium. The cultures were incubated for 24 h at 37°C with shaking at 200 rpm and the bacterial growth was monitored by measuring the OD_600_ values of the culture. All experiments include three independent replicates.

### Statistical Analysis

Statistical analysis was performed with SPSS v.22.0 (SPSS Inc., Chicago, IL, United States). Differences groups were compared using the chi-squared test and a *p*-value of <0.05 was deemed to be significant. Spearman’s rank correlation test was used for comparison of biofilm formation ability and multi-drug-resistance (MDR).

## Results

### *agr* Genotyping

By multiplex PCR, the *agr* types were successfully identified in 109 isolates, and 21 isolates were non-typeable for *agr* locus. As shown in **Table 2**, the *agr* I was most prevalent (39.2%; 51/130), followed by *agr* IV (32.3%; 42/130), *agr* II (9.2%; 12/130) and *agr* III (3.1%; 4/130). All swine farms isolates belonged to *agr* IV, whereas *S. aureus* isolated from slaughterhouse and retail indicated diverse *agr* types.

**Table 2:** The *agr* types of 130 *S. aureus* isolates from different stages of pork production.

*agr* group	Number of *S. aureus* isolates from various samples^a^			Total
	Swine farms	Slaughterhouse	Terminal markets	
I	0 (0)	15 (24.2%)	36 (92.3%)	51 (39.2%)
II	0 (0)	12 (19.4%)	0 (0)	12 (9.2%)
III	0 (0)	4 (6.5%)	0 (0)	4 (3.1%)
IV	29 (100%)	11 (17.7%)	2 (5.1%)	42 (32.3%)
*agr* (negative)	0 (0)	20 (32.3)	1 (2.6%)	21 (16.2%)
Total	29	62	39	130

^a^*The number in parentheses represents the percentage of isolates in the corresponding genotype*.

### Prevalence and Distribution of Virulence Genes

**FIGURE 1:**
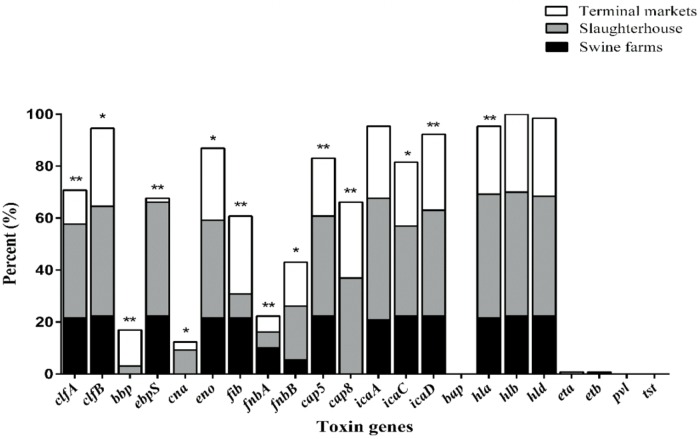
The distribution of 22 toxin genes in *S. aureus* isolates from different stages of pork production. The percentage of strains that were positive for each toxin gene was calculated based on the origin of each sample isolate. Pearson’s chi-square test (two-tailed) was used to test the difference in the virulence determinants distribution among isolates from different sources and run based on multiple group comparison of different sources. ^∗^Demonstrates that the distribution of toxin genes was statistically significantly different in isolates from different sources (*p* < 0.05); ^∗∗^demonstrates that the distribution of toxin genes was more significantly different in isolates from different food sources (*p* < 0.01).

As illustrated in **Figure 1**, nearly all isolates harbored the *hla* (95.4%), *hlb* (100%) and *hld* (98.5%) genes, encoding alpha-, beta-, and delta-hemolysins respectively. No isolate harbored *bap*, *pvl*, or *tsst*. It was found that the *bbp*, *cna* and *cap8* genes were detected only in isolates obtained from slaughterhouse and terminal markets. As shown in **Table 3**, the most frequent numbers of toxin genes per isolate were 11∼14 in all *S. aureus* isolates (**Table 3**). Notably, one isolate harbored 16 toxin genes and 5 isolates harbored 15 toxin genes, which were obtained from slaughterhouse (**Table 3**).

**Table 3:** The toxin genes number of *S. aureus* isolates from different stages of pork production.

Number of the toxin gene per isolate (*n*)	Number of *S. aureus* isolates^a^			Total number of isolates (130)^b^
	Swine farms (29)	Slaughter house (62)	Terminal markets (39)	
16	0 (0)	1 (1.6%)	0 (0)	1 (0.8%)
15	0 (0)	5 (8.1%)	0 (0)	5 (3.8%)
14	1 (3.4%)	13 (21.0%)	5 (12.8%)	19 (14.6%)
13	14 (48.3%)	9 (14.5%)	10 (25.6%)	33 (25.4%)
12	13 (44.8%)	18 (29.0%)	14 (35.9%)	45 (34.6%)
11	1 (3.4%)	5 (8.1%)	6 (15.4%)	12 (9.2%)
10	0 (0)	3 (4.8%)	3 (7.7%)	6 (4.6%)
9	0 (0)	2 (3.2%)	0 (0)	2 (1.5%)
8	0 (0)	2 (3.2%)	0 (0)	2 (1.5%)
7	0 (0)	2 (3.2%)	0 (0)	2 (1.5%)
6	0 (0)	2 (3.2%)	1 (2.6%)	3 (2.3%)

^a^*The number in parentheses represents the percentage of isolates with the corresponding number of toxin genes for all *S. aureus* isolates of the same part in pork production. ^*b*^The number in parentheses represents the percentage of isolates with the corresponding number of toxin genes for all *S. aureus* isolates*.

**FIGURE 2:**
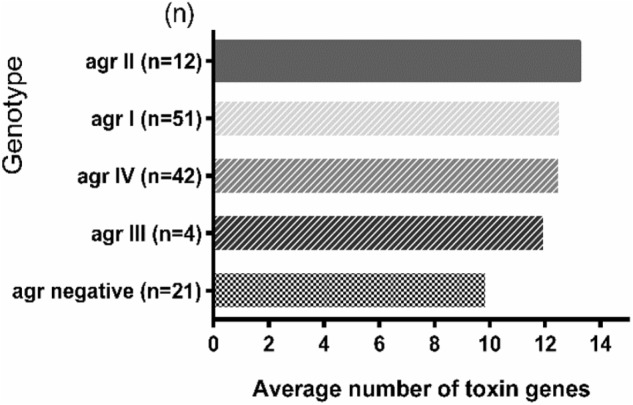
The toxin gene content of *S. aureus* isolates based on *agr* genotyping. The average number of toxin genes was calculated based on the total number of toxin genes detected in each *agr* group divided by the total number of isolates in each *agr* group. The number in parentheses on the *Y*-axis represents the total number of isolates in the corresponding *agr* group.

**FIGURE 3:**
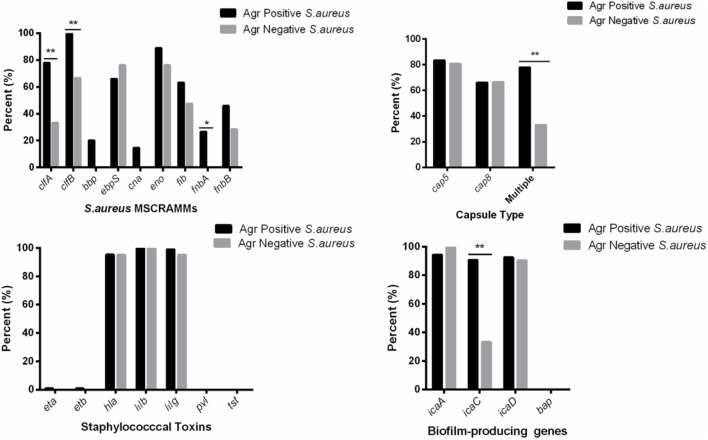
Distribution of staphylococcal virulence associated genes between *agr*-positive and *agr*-negative *S. aureus*. Pearson’s chi-square test (two-tailed) was used to test the difference in the virulence determinants distribution between *agr*-positive isolates and *agr*-negative isolates. ^∗^Demonstrates that the distribution of toxin genes was statistically significantly different in isolates from different *agr* types (*p* < 0.05); ^∗∗^Demonstrates that the distribution of toxin genes was more significantly different in isolates from different *agr* types (*p* < 0.01).

The average toxin gene number was also examined based on *agr* genotyping, and a higher average number of toxin genes was found in the *agr*-positive isolates compared to *agr*-negative isolates. The *agr*-positive isolates were associated with a high average number of toxin genes (averaging 13.2 for *agr* II, 12.6 for *agr* I, 12.6 for *agr* IV and 12.0 for *agr* III), whereas the *agr*-negative isolates were associated with a lower average number of toxin genes (averaging 9.9) (**Figure 2**). The distribution of virulence genes differed among the isolates according to the *agr* genotyping. Among the MSCRAMMs genes, the prevalence of 3 genes was significantly different between the *agr*-positive and *agr*-negative isolates: *clfA* (*p* < 0.01), *clfB* (*p* < 0.01) and *fnbA* (*p* < 0.05). The capsule multiple type (carriage of both capsule type 5 and 8) (*p* < 0.01) and *icaC* gene (*p* < 0.01) were positively associated with *agr*-positive isolates (**Figure 3**).

### Quantification of Biofilm Biomass and Growth Rate Analysis

Biofilm formation was analyzed, and all the isolates were able to form biofilm. The biomass of biofilms formed by most isolates increased continuously during incubation for 72 h at 37°C (**Table 4**). Biofilm strong producers are mainly in slaughterhouse and biofilm biomass increase with time. No significant difference in the growth rates of the strong, moderate and weak biofilm formers was observed, indicating that the difference in biofilm formation was not due to the growth rate.

**Table 4:** Biofilm phenotype of 130 *S. aureus* isolates at different time points.

		Number of *S. aureus* biofilm phenotype^a,b^								
		24 h			48 h			72 h		
Strain source	No. of strains	Weak	Moderate	Strong	Weak	Moderate	Strong	Weak	Moderate	Strong
Swine farms	29	6	21	2		13	16		2	27
		(20.7%)	(72.4%)	(6.9%)		(44.8%)	(55.2%)		(6.9%)	(93.1%)
Slaughterhouse	62	5	27	30	1	13	48		2	60
		(8.1%)	(43.6%)	(48.4%)	(1.6%)	(21.0%)	(77.4%)		(3.2%)	(96.8%)
Terminal markets	39	7	8	24	1	4	34		4	35
		(18.0%)	(20.5%)	(61.5%)	(2.6%)	(10.3%)	(87.2%)		(10.26%)	(89.7%)
Total	130	18	56	56	2	30	98		8	122
(%)		(13.8%)	(43.1%)	(43.1%)	(1.5%)	(23.1%)	(75.4%)		(6.2%)	(93.8%)

^a^*The number in parentheses represents the percentage of isolates with the corresponding number of biofilm phenotype for all *S. aureus* isolates of the same part in pork production. ^*b*^Biofilm-forming ability was measured after 24, 48, and 72 h at 37°C in terms of biofilm biomass by crystal violet staining. The results are presented by optical density (OD) determination of three independent repeats and compared to ATCC 25923 (biofilm-positive) and ATCC12228 (biofilm-negative)*.

### Correlation Between Virulence Genes and Antibiotic Resistance in Biofilm Producing *S. aureus*

**FIGURE 4:**
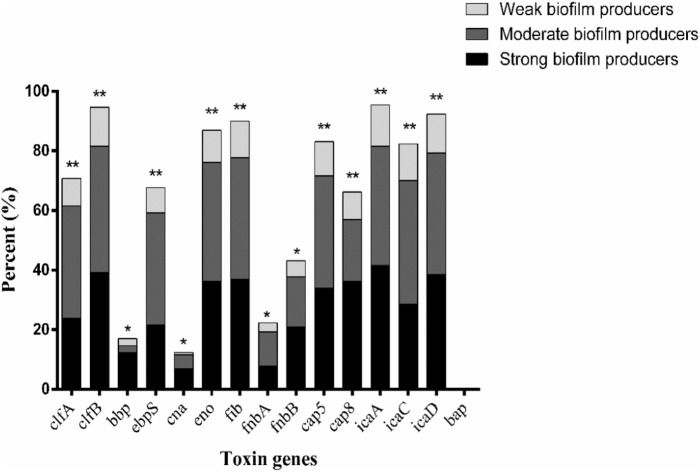
Distribution of biofilm-associated genes of *S. aureus* isolates based on biofilm formation ability. Pearson’s chi-square test (two-tailed) was used to test the difference in the virulence determinants distribution among different ability of biofilm ability. ^∗^Demonstrates that the distribution of toxin genes was statistically significantly different (*p* < 0.05); ^∗∗^demonstrates that the distribution of toxin genes was more significantly different (*p* < 0.01).

**FIGURE 5:**
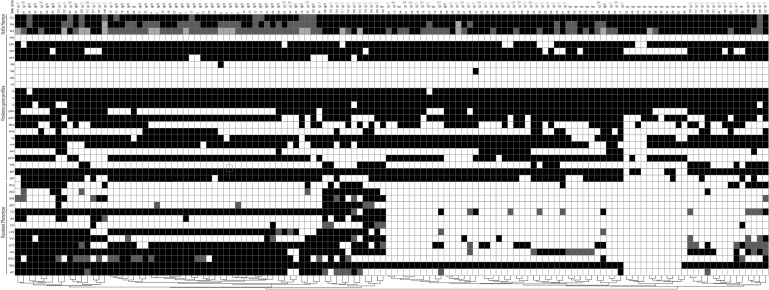
Diagram showing the antibiotic resistance pheno- and *agr* types, virulence genes profiles and biofilm phenotype of *S. aureus* isolated from different stages of pork production. The diagram was established on the basis of the presence and absence of selected determinants. For antibiotic resistance phenotype, black indicates resistance, gray indicates intermediate, and white indicates susceptible. CIP, ciprofloxacin; PEN, Penicillin; GEM, gentamicin; TET, tetracycline; CLR, clarithromycin; CHL, chloramphenicol; SXT, trimethoprim-sulfamethoxazole; NIT, nitrofurantoin; RIF, rifampicin; CLI, clindamycin; CEF, cephalothin; MIN, minocycline; OXA, oxacillin; FOX, cefoxitin; For virulence genes profiles, black indicates presence and white indicates absence. For biofilm phenotype, black indicates strong, gray indicates moderate, and white indicates weak.

The relationship between prevalence of biofilm-associated genes and biofilm formation ability (incubation for 24 h at 37°C) of *S. aureus* isolates was further analyzed (**Figures 4**, **5**). Considering the studied gene status, 19 different gene patterns were observed (**Table 5**). The most prevalent gene pattern was *clfA*-*clfB*-*ebpS*-*eno*-*fib*-*cap5*-*icaA*-*icaC*-*icaD* which was identified in 13 (10.0%) of 130 isolates. However, there was only one strong biofilm producer, nine moderate biofilm producers and three weak biofilm producers in this genes pattern. Conversely, among the genes patterns of *clfA*-*clfB*-*ebpS*-*eno*-*fib*-*fnbB*-*cap5*-*cap8*-*icaA*-*icaC*-*icaD* (3.8%,5/130), *clfB*-*eno*-*fib*-*fnbB*-*cap5*-*cap8*-*icaA*-*icaD* (1.5%, 2/130), *clfB*-*bbp*-*eno*-*fib*-*cap5*-*cap8*-*icaA*-*ic*aC-*icaD* (1.5%, 2/130), *clfA*-*clfB*-*eno*-*fib*-*fnbB*-*cap5*-*cap8*-*icaA*-*icaD* (1.5%, 2/130), and *clfB*-*eno*-*fib*-*cap5*-*cap8*-*icaA*-*icaC*-*icaD* (1.5%, 2/130), all isolates showed strong biofilm formation ability (**Table 5**). A comparison between the strong, moderate, and weak biofilm producers in the isolates showed a significant difference in the prevalence of virulence genes among these isolates.

**Table 5:** The prevalence of biofilm related genes pattern and their associations with biofilm production in 130 *S. aureus* from different stages of pork production.

Biofilm related genes patterns	Number of *S. aureus* biofilm phenotype^a^			Total
	Strong	Moderate	Weak	
*clfA*-*clfB-ebpS-eno-fib-cap5-icaA-icaC-icaD*	1	9	3	13
*clfA-clfB-ebpS-eno-fib-fnbA-cap5-icaA-icaC-icaD*	1	5	3	9
*clfA-clfB-ebpS-eno-fib-fnbB-cap5-icaA-icaC-icaD*	2	5	0	7
*clfA-clfB-ebpS-eno-fib-fnbB-cap5-cap8-icaA-icaC-icaD*	5	0	0	5
*clfA-clfB-eno-fib-fnbB-cap5-cap8-icaA-icaC-icaD*	1	3	1	5
*clfA-clfB-ebpS-cna-eno-fib-cap5-cap8-icaA-icaC-icaD*	2	2	0	4
*clfB-bbp-eno-fib-fnbB-cap8-icaA-icaC-icaD*	2	0	1	3
*clfB-eno-fib-fnbB-cap5-cap8-icaA-icaD*	2	0	0	2
*clfB-bbp-eno-fib-cap5-cap8-icaA-icaC-icaD*	2	0	0	2
*clfA-clfB-eno-fib-fnbB-cap5-cap8-icaA-icaD*	2	0	0	2
*clfB-eno-fib-cap5-cap8-icaA-icaC-icaD*	2	0	0	2
*clfA-clfB-ebpS-eno-fib-cap5-cap8-icaA-icaC-icaD*	1	1	0	2
*clfA-clfB-ebpS-fnbB-cap5-cap8-icaA-icaC-icaD*	1	0	1	2
*clfA-clfB-ebpS-cna-eno-fib-fnbB-cap5-cap8-icaA-icaC-icaD*	0	2	0	2
*clfA-clfB-ebpS-eno-fib-fnbA-cap5-icaC-icaD*	0	2	0	2
*clfA-clfB-ebpS-fib-fnbB-cap5-cap8-icaA-icaC-icaD*	0	2	0	2
*clfA-clfB-ebpS-eno-fib-cap8-icaA-icaC-icaD*	0	2	0	2
*clfB-eno-fib-fnbB-cap5-cap8-icaA-icaC-icaD*	0	1	1	2
*clfA-clfB-ebpS-fib-cap8-icaA-icaC-icaD*	0	1	1	2

^a^*Biofilm phenotype was measured after 24 h at 37°C*.

To determine whether biofilm formation was correlated with resistance to any particular antibiotic(s), we compared the biofilm forming capacities (incubation for 24 h at 37°C) among isolates with different resistance profiles for the 14 antibiotics (**Table 6**). Resistance to ciprofloxacin, gentamicin, tetracycline, clarithromycin, clindamycin and trimethoprim-sulfamethoxazole were significantly higher in moderate biofilm producers and weak biofilm producers than in strong biofilm producers (**Table 6**). Notably, resistance to nitrofurantoin was only found in strong biofilm producers (7.1%, 4/56) and moderate biofilm producers (1.8%, 1/56) (**Table 6**). Resistance to penicillin, cefoxitin and chloramphenicol showed no significant difference among strong biofilm producers, moderate biofilm producers and weak biofilm producers (**Table 6**). Regarding multidrug resistance, no significant association to strong, moderate or weak biofilm producers was observed (**Table 7**).

**Table 6:** Biofilm formation and antibiotic resistance pattern of 130 *S. aureus* isolates from different stages of pork production.

Antibiotic category	Antibiotic agent	Percentage of antibiotic-resistant strains in different biofilm phenotype		
		Strong biofilm producers (56) ^a^	Moderate biofilm producers (56)^a^	Weak biofilm producers (18)^a^
β-lactamase	Penicillin	85.7% (48/56)	98.2% (55/56)	94.4% (17/18)
	Oxacillin	17.9% (10/56)	1.8% (1/56)	11.1% (2/18)
	Cefoxitin	19.6% (11/56)	10.7% (6/56)	27.8% (5/18)
	Cephalothin	8.9% (5/56)	1.8% (1/56)	11.1% (2/18)
Fluoroquinolones	Ciprofloxacin	17.9% (10/56)	53.6% (30/56)	66.7% (12/18)
Aminoglycosides	Gentamicin	14.3% (8/56)	35.7% (20/56)	55.6% (10/18)
Tetracyclines	Tetracycline	46.4% (26/56)	58.9% (33/56)	83.3% (15/18)
	Minocycline	14.3% (8/56)	0	16.7% (3/18)
Macrolides	Clarithromycin	32.1% (18/56)	60.7% (34/56)	72.2% (13/18)
Lincomycins	Clindamycin	30.4% (17/56)	60.7% (34/56)	83.3% (15/18)
Chloramphenicols	Chloramphenicol	28.6% (16/56)	14.3% (8/56)	38.9% (7/18)
Sulfonamides	Trimethoprim-sulfamethoxazole	21.4% (12/56)	53.6% (30/56)	66.7% (12/18)
Nitrofurans	Nitrofurantoin	7.1% (4/56)	1.8% (1/56)	0
Rifamycins	Rifampicin	17.9% (10/56)	1.8 % (1/56)	27.8% (5/18)

^a^*The number in parentheses represents the corresponding number of biofilm phenotype for *S. aureus* isolates of antibiotic resistance. Biofilm phenotype was measured after 24 h at 37°C, and the number of strong biofilm producers, moderate biofilm producers and weak biofilm producers were 56, 56 and 18, respectively*.

**Table 7:** Occurrence of multidrug resistant pattern and their associations with biofilm phenotype in 130 *S. aureus* from different stages of pork production.

Number of antibiotic category	Number of *S. aureus* biofilm phenotype^a^			Total number of isolates
	Strong	Moderate	Weak	
9			1 (5.6%)	1 (0.8%)
8	6 (10.7%)	4 (7.1%)	2 (11.1%)	12 (9.2%)
7	6 (10.7%)	16 (28.6%)	6 (33.3%)	28 (21.5%)
6	3 (5.4%)	9 (16.1%)	4 (22.2%)	16 (12.3%)
5	1 (1.8%)	1 (1.8%)	2 (11.1%)	4 (3.1%)
4	1 (1.8%)	3 (5.4%)	1 (5.6%)	5 (3.8%)
3	3 (5.4%)	1 (1.8%)		4 (3.1%)
2	15 (26.8%)	7 (12.5%)	0	22 (16.9%)
1	13 (23.2%)	14 (25.0%)	1 (5.6%)	28 (21.5%)
0	8 (14.3%)	1 (1.8%)	1 (5.6%)	10 (7.7%)
Total	56 (43.1%)	56 (43.1%)	18 (13.8%)	130 (100%)

^a^*Biofilm phenotype was measured after 24 h at 37°C*.

## Discussion

The *agr* (accessory gene regulator) system is a peptide quorum-sensing system present in all the Staphylococci and a dominant regulator of pathogenesis and biofilm development in *S. aureus* (Boles and Horswill, 2008; Paharik and Horswill, 2016). All the swine farms isolates were *agr* type IV, whereas the slaughterhouse and terminal markets isolates indicated diverse *agr* types. In addition, isolates belonging to *agr*-positive group had a higher number of toxin genes than those belonging to *agr*-negative group (*p* < 0.05), suggesting that *agr* profiles may be associated with the virulence potential of *S. aureus*, which is consistent with a previous finding (Cheung et al., 2011). Raw meat isolates (belonging to *agr* I) exhibited a great ability to form strong biofilms than swine farms isolates (belonging to *agr* IV). Previous studies have shown that biofilm formation in *S. aureus* isolated from bovine mastitis with *agr* I is higher than those with other *agr* types (Bardiau et al., 2013; Bardiau et al., 2014; Khoramrooz et al., 2016).

The prevalence of virulence genes involved in biofilm formation and staphylococcal toxin genes were investigated. Most biofilm-producing isolates were positive for MSCRAMM, capsule type and *ica* group genes. The data show a significant association between the prevalence rate of MSCRAMM, capsule type and *ica* group genes among isolates producing weak, moderate and strong biofilms. Approximately 92.3% (120/130) of all isolates harbored *icaA* and *icaD* genes simultaneously, which were similar to those from previous studies (Szweda et al., 2012; Pereyra et al., 2016). Moreover, although both *pvl* and *tst* genes were not detected in the tested isolates, hemolysins and enterotoxin-producing genes (data not shown) were found. This suggests that these isolates exhibit pathogenic potential.

In the present study, all *S. aureus* isolates were biofilm producers. Biofilm formation is influenced by numerous factors, such as sugar content and concentration (glucose versus lactose), proteolytic enzymes and biofilm-associated genes, etc. (Coelho et al., 2008). In this study, biofilm production was higher for raw meat isolates compared to swine farms isolates. There was a difference in the prevalence of several genes involved in adhesion and biofilm production between raw meat and swine farms isolates. However, further studies are required to quantify the expression of relevant genes. Moreover, biofilm biomass increased proportionally as biofilms aged, which is accordance with previous findings (Akinbobola et al., 2017). High variability in biofilm biomass was found among isolates throughout the time course of biofilm formation (24 – 72 h), which is in accordance with previous findings (Marino et al., 2011; Va′zquez-Sa′nchez et al., 2014). Moreover, our study demonstrated the potential association between antibiotic resistance and biofilm-forming ability of *S. aureus*. Apart from resistance to penicillin, the high prevalence of resistance to ciprofloxacin, gentamicin, tetracycline, clarithromycin, clindamycin and trimethoprim-sulfamethoxazole were mainly observed in moderate and weak biofilm producers. Together, Qi et al. (2016) reported that for *Acinetobacter baumannii*, there was a statistically negative correlation between antibiotic resistance and biofilm forming capacity, suggesting that biofilm-forming strains are less dependent on antibiotic resistance than no biofilm-forming strains for survival. Previous studies have demonstrated that biofilm resistance to antimicrobials is multifaceted, including reduced penetration of the agent into biofilms due to the presence of extracellular matrix, biofilm heterogeneity and biofilm-specific phenotypes such as expression of efflux pump and persister cells (Stewart and Costerton, 2001; Akinbobola et al., 2017). Moreover biofilm resistance is known to vary from one microorganism to another (Mah and O’Toole, 2001). Thus our further study will focus on the enhancement in resistance of our *Staphylococcus aureus* after biofilm formation.

In summary, our study revealed *agr* type diversity, virulence potential, antibiotic multiresistance and high biofilm formation ability of *S. aureus* isolated from pork production. All swine farms isolates belonged to *agr* IV, whereas *S. aureus* isolated from slaughterhouse and retail indicated diverse *agr* types. Raw meat isolates (belonging to *agr* I) exhibited a great ability to form strong biofilms than swine farms isolates (belonging to *agr* IV). Most biofilm-producing isolates were positive for MSCRAMM, capsule type and *ica* group genes. The results illustrate a significant association between the prevalence rate of MSCRAMM, capsule type and *ica* group genes among isolates producing weak, moderate and strong biofilms. Clarifying these mechanisms could provide novel insights that would prevention against *S. aureus* biofilm-related infections.

## Author Contributions

HY and LS participated in the design of this study. RC, DX, LS, and CL provided assistance for concepts, design, literature search, data acquisition, and manuscript preparation. YZ collected important background information, carried out the study, and performed the statistical analysis. HY and YZ drafted the manuscript. HY and DX performed the manuscript review. All the authors have read and approved the content of the manuscript.

## Conflict of Interest Statement

The authors declare that the research was conducted in the absence of any commercial or financial relationships that could be construed as a potential conflict of interest.

## Footnotes

1 www.ncbi.nlm.nih.gov
